# Exploring grant writing coaching and its role in professional development of health equity investigators: A qualitative study

**DOI:** 10.1017/cts.2025.10080

**Published:** 2025-07-01

**Authors:** Yulia A. Levites Strekalova, Selin Kavak, Vanessa Caridad Rodriguez, Sara Midence, Lee S. Caplan, Winston Thompson, Muhammed Y. Idris, Jonathan Stiles, Priscilla Pemu, Alexander Quarshie, Adriana Baez, Maritza Salazar Campo, Elizabeth Ofili

**Affiliations:** 1 University of Florida Clinical and Translational Science Institute, Gainesville, FL, USA; 2 Department of Health Services Research Management and Policy College of Public Health and Health Professions, University of Florida, Gainesville, FL, USA; 3 Morehouse School of Medicine, Atlanta, GA, USA; 4 Department of Pharmacology and Otolaryngology-Head and Neck Surgery, School of Medicine, University of Puerto Rico, San Juan, PR, USA; 5 Paul Merage School of Business, University of California Irvine, Irvine, CA, USA

**Keywords:** Coaching, grant writing, mentoring, social support, workforce development

## Abstract

**Introduction:**

External funding is a critical metric in research career advancement, particularly in biomedical fields. Grant-writing coaching emerges as a strategy in biomedical workforce development. Recognizing disparities in grant success among early-career investigators from underrepresented groups, the National Research Mentoring Network Strategic Empowerment Tailored for Health Equity Investigators (NRMN-SETH) provides grant-writing coaching to support these scholars. This study explores the roles of NRMN-SETH grant-writing coaches in fostering technical skills and social support in a group setting.

**Methods:**

This qualitative study employed semi-structured interviews with 16 NIH-funded investigators who served as coaches within the NRMN-SETH program. Data were transcribed, coded, and analyzed using the Framework Method, identifying key roles related to coaching practices.

**Results:**

Findings reveal that grant-writing coaching involved personalized guidance, confidence-building, and structured group interactions. Coaches emphasized individualized feedback on grant components and provided iterative guidance. The group-based coaching environment fostered peer support and normalized challenges, creating a collaborative atmosphere conducive to skill-building. Coaches noted the importance of institutional support in enabling participants to engage in the program, though challenges arose in managing participants with varying grant-writing experience.

**Conclusions:**

This study highlights the potential of grant-writing coaching to enhance research capacity among underrepresented scholars, offering a structured, supportive approach that complements traditional mentorship. Integrating tailored coaching programs within biomedical workforce development, particularly at minority-serving and low-resourced institutions, may reduce disparities in grant success. Future research could expand on these findings by investigating the long-term career impacts of coaching and testing the effectiveness of peer-led, group-based components in grant-writing success.

## Introduction

External funding is a key marker for both institutional and individual success in biomedical research. There has been a concerted effort to integrate grant writing coaching into the professional development programs offered to faculty and researchers to support grant writing skill development, which requires both the technical knowledge about grant proposals, their components, and submission formats and a supportive ecosystem of senior and peer colleagues who provide coaching, encouragement, and accountability throughout the process of grant development. Grant writing support programs often include workshops, one-on-one and group coaching sessions, and peer review processes designed to improve the quality of grant submissions. The growing popularity of these initiatives reflects a broader trend toward professionalizing the process of grant writing and recognizing it as a distinct skill set that requires dedicated training and support [1]. While mentorship has been a cornerstone of academic career development, coaching has emerged as a distinct and complementary tool for academic professional development [2,3]. Next, we will discuss the evolving roles of mentors and coaches in biomedical workforce development.

### The evolving role of mentorship in academic development

Traditionally, mentorship has been associated with developing research skills, personal growth, and career advancement. The literature underscores the importance of mentoring in increasing research productivity, enhancing grant success, and promoting the overall development of junior faculty [4]. Mentorship also supports personal development, as it provides mentees with the necessary tools to overcome the challenges of academic career progression and fosters a sense of belonging within the academic community [4].

Recent studies and academic development practice have expanded the concept of mentorship to include various models, such as peer mentoring and group mentoring, which address the diverse needs of early-career investigators. These models recognize that traditional one-on-one mentorship may fall short in addressing today’s complex and multifaceted challenges in academia. For instance, group mentoring allows mentees to benefit from the collective wisdom of multiple mentors [4, 5] or interact with one mentor and several peers [6], thereby developing a professional development community and a support network. This evolution in mentoring practices reflects a broader understanding of the dynamic nature of academic careers and the need for adaptable and responsive mentoring frameworks.

### Coaching as a tool for academic development

Mentoring and coaching are both pivotal in academic professional development, though they serve distinct purposes and are often confused or conflated. Mentoring is a development activity that significantly influences a mentee’s professional growth, usually characterized by a long-term, holistic relationship aimed at career advancement and skill development [7]. In contrast, coaching is commonly short-term and goal-oriented, focusing on specific tasks and skills enhancement [8]. Both mentoring and coaching share overlapping skills, such as providing feedback and guidance; however, mentoring tends to be broader and more relationship-oriented, whereas coaching zeroes in on performance improvement in particular areas [9]. Furthermore, the context in which these activities occur can deeply influence their effectiveness [9]. Mentoring and coaching are increasingly combined to leverage both methodologies’ strengths, offering more flexible and comprehensive development opportunities within academic settings [10]. Unlike mentoring, which is typically characterized by long-term relationships and broad career guidance, coaching focuses on specific tasks or skills and aims to improve performance in a particular area [11]. Coaching is often iterative, involving observation, feedback, and re-performance cycles designed to help the coached achieve predefined goals. This approach is particularly effective in developing technical skills and has positively impacted physician well-being, making it an essential tool in the academic development of biomedical professionals [4].

Grant writing coaching has emerged as a critical component of academic development, particularly for early-career investigators seeking to secure funding in an increasingly competitive environment [12, 13]. The literature highlights that coaching is about improving isolated skills and fostering a mindset of continuous improvement and self-reflection. This approach has rapidly gained recognition as a structured and effective means of enhancing investigators’ abilities to craft compelling grant proposals. Unlike traditional mentoring, which tends to be broader in scope, grant writing coaching focuses specifically on the skills and strategies necessary for successful grant applications. The structured nature of coaching provides targeted support tailored to each researcher’s unique needs, making it a valuable tool in the academic development toolkit [14]. Through regular and structured feedback, coaching helps individuals refine their approach to specific challenges, enhancing their overall professional effectiveness. This is particularly relevant in academic medicine, where the demands for clinical and research excellence are high. By focusing on targeted areas of development, coaching complements the broader scope of mentoring, providing a more nuanced support for early-career investigators [15].

One of the most compelling reasons for the growing emphasis on grant writing coaching is its potential to address disparities in grant success, particularly among underrepresented groups in biomedical research. Research has shown that minority and female researchers often face systemic barriers that limit their participation in funded research [16–20]. Grant writing coaching can play a crucial role in leveling the playing field by providing these investigators with the support they need to overcome these predictable barriers and unexpected setbacks [21]. For example, tailored coaching programs that focus on the challenges faced by underrepresented groups have been shown to increase their success rates in securing grants [22]. Adding coaching to the arsenal of workforce development tools enhances the opportunities for early-stage investigators to access broad and diverse perspectives and supports [23].

The efficacy of group-based coaching for academic professional development has been supported previously. Group coaching promotes collective problem-solving, allows participants to navigate group dynamics, and helps create peer learning communities to foster mutual support in organizational settings [24]. Furthermore, group coaching using video-conferencing was shown to reduce burnout and promote well-being among early-career researchers by providing emotional and professional support [25]. Specific to grant writing, group coaching provides participants with how iterative feedback and engagement with expert colleagues in group coaching contexts significantly enhance grant proposal writing among biomedical researchers [26]. This approach promotes clarity in writing and improves the quality and precision of proposals through comprehensive peer feedback [26].

### Goal of the present study

The evolving practice of mentorship and coaching in career development highlights the need for a multifaceted approach to supporting early-stage investigators. Grant writing coaching is a rapidly developing and increasingly essential competence-based strategy for academic career development. Its impact on research productivity and its potential to address disparities in grant success make it a valuable tool for researchers and institutions. Still, the roles of the coaches lack clarity. This study aimed to address this gap and answer the following research question:

*What are the mechanisms through which grant writing coaches provide technical and social support to early-stage biomedical investigators?*

## Materials and methods

### Study setting

The study reported here is part of a larger initiative within the National Research Mentoring Network (NRMN), a National Institutes of Health (NIH)-funded initiative designed to enhance the diversity of the biomedical research workforce by providing mentorship, professional development, and grant-writing coaching to early-career investigators. The NRMN aims to support individuals from underrepresented groups in biomedical research, including those at minority-serving institutions (MSIs) and other low-resourced institutions. Central to the NRMN’s mission is the provision of grant writing coaching, a key component designed to equip early-stage investigators with the skills necessary to secure research funding and build successful research careers. The NRMN’s approach to grant writing coaching is structured and multifaceted, encompassing individualized coaching, group-based learning, and access to a network of experienced mentors. The focus is on building participants’ confidence and competence in grant writing, with particular emphasis on aligning their research proposals with funding priorities and navigating the complexities of the grant submission process. By fostering these skills, the NRMN not only enhances the grant writing abilities of participants but also contributes to their overall professional development and research productivity [27].

Within the NRMN network, the Strategic Empowerment Tailored for Health Equity Investigators (NRMN-SETH) award includes supporting investigators from minority-serving institutions (MSIs) and other low-resourced institutions. The NRMN-SETH aims to address the disparities in grant funding success among researchers from underrepresented groups by providing targeted grant writing support and fostering an inclusive research environment. The barriers to success can include a lack of access to experienced mentors, limited research infrastructure, and the challenges of balancing heavy teaching loads with research activities [28, 29]. The NRMN-SETH addresses these challenges by providing tailored coaching that helps investigators develop the skills to write compelling grant proposals that compete successfully for NIH and other funding. Specifically, the program supports investigators through a combination of grant writing workshops, group coaching, and peer mentoring. These activities are tailored to address the unique challenges researchers face at MSIs and low-resourced institutions, such as limited access to research infrastructure and mentorship opportunities. By providing these researchers with the tools and support necessary to develop competitive grant proposals, the NRMN-SETH award plays a crucial role in enhancing the diversity of the biomedical research workforce [30, 31]. The NRMN-SETH initiative also focuses on creating sustainable research environments at these institutions by fostering collaborations and building research capacity [32]. This collaborative approach is particularly important for minority-serving and low-resourced institutions, where resources may be limited, and the collective strength of a research network can significantly enhance the likelihood of grant success [21, 33, 34].

### Study design and review

This qualitative study employed a semi-structured interview design. Interviews aimed to elicit the experiences of established NIH-funded investigators who served as coaches for the NRMN-SETH project. The study protocol was submitted for review to the Institutional Review Board of the first author and received an exempt designation. All participants provided verbal consent for the recording and use of the interview data for analyses.

### Participants

NRMN-SETH program had five cohorts, with most coaches participating in more than one cohort, and all NRMN-SETH coaches were invited to participate in interviews. All coaches have had prior successful experience with NIH funding and represented a diversity of institutions (e.g., minority-serving, very high (R1), and high (R2) research activity). Coaches received a small honorarium for the coaching activities, but no compensation was offered for the interviews. All coaches were introduced to established practices in group-based grant-writing coaching [35] and encouraged to integrate it with their own approaches to proposal development.

### Data collection and analysis

Experiences of coaches and elicitation of coach roles were explored using semi-structured, one-on-one interviews. The interview guide (Appendix A) was developed in consultation with the NRMN-SETH program staff and piloted with grant writing coaches from an institutional program of the first author. Audio recordings were transcribed using secure automatic transcription tools and checked for correctness, after which the audio files were deleted. Data were managed using the Framework Method [36]. The first author read the transcripts and developed an initial coding framework, which was applied to all transcripts by two additional coders. Additional emerging codes were discussed within the team and existing codes were modified to incorporate newly identified items. The coded text was reduced to create a participant-by-theme matrix illustrating key points and providing key illustrative quotes from each transcript.

## Results

The content analysis of responses from the 16 coaches reveals several recurring topics that reflect the collective experience and insights of the coaches in the NRMN program. Multiple coaches address these topics, indicating common activities in grant writing coaching related to (1) providing personalized guidance, (2) managing group dynamics, (3) navigating grant writing capacity, and (4) extending support beyond formal meetings (see Table 1 for the themes, subthemes, and representative quotes).

**Table 1.** Identified themes, subthemes, and representative quotes of coaching activities

Theme	Subtheme	Representative Quotes
Provide Personalized Guidance	Offer individualized feedback	We’re based on each trainee’s need for a plan we can tailor. You know, and we individualize the program to make sure they’re making progress to the submission of their grant. (NRMN_16)
	Tailor guidance to meet participants’ needs	I went back and forth with them on their specific aims over and over again, and essentially said, “You know, once you get your specific aims really nailed down the rest of the proposal is gonna largely write itself.” And that, that took a long time. (NRMN_01) The meetings were driven by them. This is not dealing with students; this is dealing with active professionals who have obligations and who really are doing this as something outside their normal, everyday roles. None of them was employed as a grant writer to just write grants. And so, I was extremely flexible. (NRMN_01)
	Create a safe environment to build confidence	So when we meet, they know there’s a trust first of all, and there’s dedication for the others. So if you are dedicated to the others, the others are dedicated for you, and I’ve seen a lot of interaction that way. Especially for the ones that stay until the end. They would even call each other behind, you know, behind the lines to help each other. […] They’ll be working with each other during the time in between meetings. So I think the coffee culture, if you want, […] or kind of trust. It’s like friends coming along every 2 weeks to have coffee and talk about their struggles, and see how we can help. (NRMN_09) So our routine in the meeting was to check in with each other, and just a mini kind of 5-minute like, how are folks doing? And folks were juggling a lot. They were stressed. There’s a lot of burnout even among early career faculty. We would do a little bit of check-in and support for one another. (NRMN_06)
Manage Group Dynamics	Set regular group meetings	You have structured meetings, and you’re working together as a team. (NRMN_11) In the beginning, it was more intense meetings, but when the group was large, it became very hard. I think, on average, we were meeting like every other week to the end. But when the group is more than 4 you meet for 2 hours every other week, but that 2 hours is not enough for the 8 or 7 that you have. So we had to interlace weeks [to] be weekly for a certain period of time until they become independent. Then we space it out again every 2 weeks. (NRMN_09) I think our group accomplished energy and synergy with one another. There was a core group of people we met every other week for a 2-hour block, I think. And most weeks most people were there. And so I think we accomplished kind of a right - I think people came because they found it useful. (NRMN_06)
	Establish milestones and deliverables	“For every meeting we had a list of deliverables to work on. So, for example, for one meeting, we would say, we’re all gonna work on the significant section, and then we’re going to upload that to Slack and review it before the meeting, and then go over each of your work, and you know, give feedback from me as well as from each other. And then we set the next milestone for the next meeting. So like, next, we’re gonna work on innovation. And essentially, that’s how it went.” (NRMN_15). I think I got better in the second cohort at kind of helping people understand and actually putting together a timeline and a spreadsheet. So here’s all our meetings. Who’s gonna submit what for what meeting? And we would work backwards from the due date of their grant. So that we say, “Okay, you need to have your specific aims for this.” And so we all had sort of a timeline to work from, and we knew what to expect from whom and when, and that helped us in planning. (NRMN_05)
	Check in and maintain engagement	I would just usually, before the meeting, send a reminder, and then just say, you know, here are some possible things to talk about today. (NRMN_10) I would communicate through Slack. So, after each meeting I would say, Hey, just a reminder for our next meeting, we decided we’re going to prepare this portion of the grant; and then […] a couple of days before [the meeting] I would send them another reminder, “Please remember to upload your work,” and they would usually upload it the same day. [and] when they couldn’t make it, they would communicate through slack, and I can provide them some written feedback through Slack. (NRMN_15) Some would not show up, cancel very last minute, or say they’re not ready to share things. (NRMN_07) I insisted on sticking to the commitment despite shifting priorities. (NRMN_09)
	Facilitate peer-to-peer review	During the meeting, everybody [would] share a screen and go through their proposal, and I give feedback, and then the participants give feedback. [..] it approximated to them a little bit the process that the reviewers go through because, essentially, they just read the proposal a couple of times and write down their feedback in the form of a critique. (NRMN_15)
Navigate Grant Writing Capacity	Assess individual grant writing readiness	Some of them were not as advanced in their writing as others so, even though they came to meetings sometimes they would not really progress in their writing. And it was hard because they all have different schedules and other responsibilities and also based on their own career stage and how much, I guess, mentorship they get in real life, so for some of them with those harder to write. (NRMN_15) Some of the times people are not ready to and they will subscribe to the program, the mentee, I mean without being ready and I get them. And they would have a proposal that is 10 years old. They haven’t looked at it. I have to - you know, work harder with them, and sometimes, you know, they would feel by themselves that they are not ready, and they will disengage early or midway, or something like that, for those that stay usually are those that are really there to have an outcome. (NRMN_09) The participants in terms of grant writing, sometimes, I think one or one or 2 participants actually is not in that level is basically we are doing the guiding in terms of a manuscript writing instead of grant writing, because she doesn’t. As far as I recall, she haven’t had any experience in terms of grant writing, and also is not ready. I mean, she doesn’t have a topic. There’s nothing we can do about. I mean I cannot put my topic into her head. (NRMN_07) So people even didn’t have a project. And they wanna write a grant. So that was like and then they stop. Some of them stop attending the meeting throughout. Some of them, I think maybe dispirited. At least one person out that I have in the 2 cohorts was, I think one person also thought maybe even so dispiriting for that individual because they didn’t have a project or was so establishing their lab. (NRMN_14) I think maybe having somebody in the room with more expertise in that area would have benefited the conversation. (NRMN_13)
	Encourage participation in mock study sections	You know how eye-opening that was, and again, I think that was something that the program offered that some of them didn’t take advantage of, and I think that was a shame. I think that would have been a great opportunity for them to get very good constructive feedback. (NRMN_13) I attended the mock session, and some of the feedback was similar to the things that was telling them. There were some new insights. […] I think it’s critical. I think it’s important that they see the wide variety of comments that come or based on the backgrounds of the people who we’re reviewing the grant. (NRMN_08)
	Emphasize institutional and programmatic support	I think they would need to have buy-in from the institution to provide a time dedicated for on both sides, both mentors and mentees, who like really invest, you know, their effort into the program. I think that that’s an important… I would, you know, want the colleges also buying, or just like the institution, and have support for, for faculty to participate. (NRMN_13) I think that any similar program would need institutional support to actually free up the faculty time required for mentoring and make sure mentees can take advantage of the program. I mean, mentoring is important, but without dedicated time for it, it’s really difficult. (NRMN_02)
Extend Support beyond Formal Meetings	Provide accessibility for urgent assistance	I met formally every other week. But some of my mentees, they actually sent me emails 3 times, sometimes 4 times a week. I provided them my cell phone number. If there is emergency to have an urgent question, if I can help them with their grant application, they could call me call my office or call me. (NRMN_14) […] with each one of them, we kept having conversations and email exchanges and feedback exchanges offline. And then we just started scheduling individual meetings in the spring semester. (NRMN_04) I really felt like I got to know these early career investigators, and I’m happy that some of us have stayed in touch, and that they’re doing well and getting some of their grants funded and moving ahead in their careers. (NRMN_05)
	Consider support beyond the coaching program	[T]he last time I contacted with a cohort, I talked to a member of my cohort 2; it was like 3 weeks ago. She […] wanted to submit a grant to a small foundation, and she needed a support letter. So, of course, I wrote her a support letter. (NRMN_14) […] the person who submitted a K. Award was really, I thought, was a really good example. I do not think she would have submitted it without this program. You know, as a family with young kids, [she] was visibly sometimes stressed, but continually made progress. (NRMN_04)

### Provide Personalized Guidance

Coaches viewed personalized guidance as one of the core grant writing coaching activities, which included the need to *offer individualized feedback*, *tailor guidance to meet program participants’ needs*, and *create a safe environment to build confidence*. Many coaches emphasized the importance of providing individualized feedback and coaching tailored to the specific needs of each participant. While standard coaching sessions had a group format, coaches often met one-on-one with the participants to address their unique challenges “*to make sure they are making progress*” (NRMN_16). This personalized approach was central to helping participants navigate the complexities of grant writing, offering them the guidance needed to overcome specific challenges and improve their proposals. The coaches often tailored sessions to address the unique concerns of each participant, providing detailed feedback on documents, structuring grant proposals, and offering advice on various aspects of the grant writing process.

Coaches emphasized that they often tailored guidance to meet participants’ needs. In describing the process of reviewing drafts and proposal components, one coach shared how they met with participants repeatedly to refine their specific aims. While working on the specific aims “over and over again” took a long time, the coaches offered encouragement to the participants that once the “*specific aims [are] really nailed down, the rest of the proposal is gonna largely write itself*” (NRMN_01). This iterative process was key to ensuring that the proposals were well-structured and clearly articulated. Another coach also highlighted the detailed attention given to participants’ work, noting, “*I spent a long time on methodology*” (NRMN_03) underscoring the importance of thoroughly reviewing each section of the grant proposal to ensure clarity and rigor. While following a logical progression in guiding the development of proposals, the coaches demonstrated flexibility in their support by being responsive to the specific timing needs of participants. Coaches often adjusted communication methods to fit the situation and shared how they provided support to help participants stay on track, even when they were facing challenges, highlighting how continuous encouragement was a key aspect of their coaching approach. The coaches stayed flexible to give the participants agency and ensure that “*the meetings were driven by them* [participants].” (NMRN_01).

#### Create a safe environment to build confidence

Coaches reflected on how they worked closely with participants to develop their confidence in grant writing by viewing program participants as junior colleagues, aiming to build rapport and confidence and create a supportive and collaborative coaching environment. Confidence building included both the attention to technical and social aspects of grant writing. On the technical side, this included reviewing documents, structuring grant proposals, and offering guidance on various aspects of grant writing. Coaches also focused on demystifying the process by sharing examples and models to follow: “I was able to share examples with them of full proposals that I might not have had otherwise.” The coach also prompted future coaching programs to consider some questions: “[*W]ere they able to see other examples? Did they have access to things like that? I think that’s incredibly valuable to have […] other examples of successful applications*.”(NRMN_10).

In terms of the social aspect of confidence-building, coaches viewed informal social interaction as a foundational aspect of building trust, accountability, and “*dedication for the others*” (NRMN_09). Proactive communication helped participants stay on track with their writing goals, but the group format of the program helped normalize the different challenges that participants faced along the way. Coaches recognized that “*[t]here’s a lot of burnout even among early-career faculty*” (NRMN_06), and nurtured the social support network to promote social connection and self-efficacy among the participants.

### Manage group dynamics

#### Set regular group meetings

Coaches carried out the activity of organizing group work and set meetings every 2-3 weeks for their groups. While the frequency of the meetings was dictated by the NRMN-SET program, the coaches acted as the core facilitators for group interactions. These regular meetings allowed for consistent progress and the ability to “*work together as a team*” (NRMN_11) to address issues as they arose. Although the NRMN-SETH participants are asked to commit 10 hours or more a week to grant writing, finding a recurring time that works for several people requires some negotiation and adjustment. Coaches described the individualized attention they provided by scheduling bi-weekly meetings and offering continuous support. While most coaching groups had 4-6 participants, some groups were larger due to the group randomization assignment. The larger group size created logistical challenges and required coaches to “*interlace weeks [to] be weekly for a certain period of time”* (NRMN_09) before switching to less frequent meetings. For most coaching groups, the meetings took about two hours. While this time was focused mostly on technical work, structuring drafts, and getting comments, the coaches also noted that these regular meetings served the social function of building a supportive peer community for the “*core group*” of participants who attended the sessions regularly. (NRMN_06)

#### Establish milestones and deliverables

Following the program framework, coaches implemented a structured approach by setting clear milestones and “*a list of deliverables”* (NRMN_15) for each meeting, ensuring that participants made consistent progress toward their goals. Coaches discussed specific grant sections as development milestones and worked to ensure that participants set and adhered to the agreed-upon schedule. Coaches identified this structured approach as a mechanism for both progress and accountability for program participants. Regular meeting schedules helped to create a consistent rhythm, but coaches also noted the need to develop the means for open accountability and planning. Over time, as coaches became more familiar with their role, they developed tools to define and track the specific tasks the participants needed to complete by certain dates. As one coach put it, “*I think I got better in the second cohort at [] helping people understand and actually putting together a timeline*” (NRMN_05).

#### Check in and maintain engagement

In addition to setting up regular meetings, coaches emphasized the importance of holding participants accountable to the agreed-upon schedules through regular follow-ups and reminders. Coaches viewed this accountability mechanism as a crucial element of the coaching process, ensuring that participants adhered to the schedules and completed their assigned tasks. Most coaches took it upon themselves to send reminders as a tool to maintain accountability by establishing clear milestones, regularly following up on participants’ progress, ensuring that participants were prepared and aware of upcoming tasks, and “*insist[ing] on sticking to the commitment despite shifting priorities*” (NRMN_09). Coaches highlighted the challenge of getting all participants to contribute equally as the level of engagement among participants varied. Some participants were highly proactive, actively seeking feedback and participating in discussions, while others struggled to stay on track with their tasks, *“would not show up, [] or say they’re not ready to share things*” (NRMN_07). This variability created challenges in maintaining a productive group dynamic and ensuring that all participants benefited equally from the coaching process. Another coach experienced similar challenges, stating, “*Some people didn’t show up; it’s hard to follow*.” (NRMN_14) This inconsistency in participation affected the overall effectiveness of the coaching process. This lack of consistency was another role for the coaches who realized the need to maintain momentum and group cohesion.

#### Facilitate peer-to-peer review

Peer-to-peer review is an essential part of the NRMN-SETH grant coaching experience. Many coaches commented on their experience facilitating the peer-to-peer review and collaborative feedback, and coaches also described their efforts to host discussions around grant writing and career development, saying, “*I facilitated the group providing feedback*” (NRMN_05). Coaches discussed the benefits of real-time peer feedback, noting that it helped participants understand the review process. This approach helped to create a supportive environment where participants could learn from each other’s experiences. However, coaches observed that peer interactions were sometimes limited by the participants’ varying levels of experience and noted that the success of this approach depended on the group’s dynamics, with some groups benefiting significantly from the interactions, while others struggled to engage in meaningful discussions: “*I tried to figure it out and encourage them to share and request feedback, but I wasn’t very successful*.” (NRMN_13) Finally, coaches noted that they had to intentionally push for discussions during meetings: “*I intentionally forced them to have discussions during the meeting*.” (NRMN_16) This effort was crucial for ensuring that participants engaged with each other and benefited from the collective knowledge of the group.

### Navigate grant writing capacity

#### Assess individual grant writing readiness

Coaches frequently encountered participants with different levels of experience in grant writing. This created challenges in providing appropriate guidance, as some participants were well-prepared to submit grants, while others were still developing the foundational skills needed to draft a competitive proposal and “*were not as advanced in their writing as others* ” (NRMN_15). This variability in experience often made it challenging for coaches to deliver guidance that was equally beneficial to all participants. Some coaches suggested that participants should have prior grant submission experience to benefit fully from the program. This would ensure that participants have a foundational understanding of the grant writing process.

One coach described the difficulty in maintaining cohesion within a group with varying experience levels, when “*people are not ready*” or “*they would have a proposal that is 10 years old*” (NRMN_09). These issues were sometimes exacerbated by the participants’ inexperience with grant writing or not having a well-articulated research topic, leading to frustration and disengagement. The differing levels of progress in grant writing made it challenging to keep all participants aligned. This created a situation where more experienced participants could move forward while those less familiar with grant writing struggled to keep up. One coach mentioned the struggle of working with mentees who were not ready to submit a grant. This lack of preparedness made it challenging to provide effective coaching. Several coaches suggested that participants should have prior experience in grant submission before entering the program. The need for prior experience emerged as a potential solution to these challenges, with coaches advocating for a more selective entry process that would ensure participants are adequately prepared to benefit from the coaching program. This prerequisite would ensure that participants have a basic understanding of the grant writing process, allowing them to gain more from the coaching provided.

#### Encourage participation in mock study sections

The mock study sections were viewed as a valuable component of the program, providing participants with critical feedback and a realistic experience of the grant review process. Mock study sections also extended the group experience to a larger ecosystem for the reinforcement of progress and accomplishments. However, not all participants took advantage of the mock study sections, which limited their exposure to constructive criticism and the opportunity to improve their grant proposals. Coaches viewed the mock study sections as a valuable and realistic component of the program, providing participants with a critical experience that mimicked the formal review process of grant proposals. Many coaches perceived these sessions as essential for helping participants understand the rigor and expectations of actual funding reviews, with some describing them as *eye-opening* opportunities that “*some of them [participants] didn’t take advantage of*” (NRMN_13).

In preparing participants for the mock study sections, coaches played a pivotal role in encouraging a mindset of readiness and resilience. They frequently guided participants through the grant’s individual sections, helping them anticipate questions and critiques they might receive. Coaches often dedicated additional one-on-one time or small group sessions to refine specific aims, research design, and presentation of the proposal’s significance. They emphasized clarity and coherence, urging participants to present their ideas in ways that would be compelling to reviewers. By providing detailed feedback and encouraging revisions, coaches helped participants polish their proposals and gain confidence in presenting their research ideas. Furthermore, coaches helped participants prepare emotionally, normalizing the rigorous nature of the review process and encouraging them to view critical feedback as a learning tool rather than a setback. Coaches often reminded participants that such feedback was part of the process, reassuring them that constructive critiques would ultimately strengthen their proposals. Through this preparation and encouragement, coaches fostered a supportive environment, enabling participants to approach the mock study sections with a more robust and open perspective, ultimately readying them for real-world grant evaluations.

#### Emphasize institutional and programmatic support

The success of the NRMN program was heavily influenced by the level of institutional and programmatic support and “*buy-in*” (NRMN_13) available to both coaches and participants. Coaches frequently highlighted the need for strong institutional buy-in and support to ensure that participants could fully engage with the program. This support was crucial in providing participants with the protected time needed to focus on their grant writing and in integrating the program into the broader institutional framework, thereby enhancing its effectiveness and sustainability. Coaches noted that without robust institutional support, participants are likely to struggle with the demands of the NRMN program. Therefore, institutional buy-in was viewed as critical for the success of programs like NRMN, as it allows participants to engage deeply with the coaching process and take full advantage of the resources and guidance provided.

Coaches emphasized that for the NRMN program to be truly effective, there must be a strong commitment from the participants’ home institutions to “*institutional support to actually free up the faculty time required for mentoring*” (NRMN_02). This commitment includes providing protected time for participants to focus on grant writing, offering necessary resources, and recognizing the effort of the coaches and the potential contribution of coaching programs for the institution’s overall research infrastructure. To capitalize on the value of grant writing coaching programs, the institutions need to provide the necessary time, resources, and infrastructure to support their faculty and staff in their grant writing endeavors. This support not only helps participants focus on developing high-quality grant proposals but also ensures that the program’s benefits are fully realized within the broader context of the institution’s goals.

### Extend support beyond formal meetings

#### Provide accessibility for urgent assistance

Coaches across the program described a strong commitment to accessibility and responsiveness, emphasizing that being available for participants was integral to an effective coaching process. They often extended their support beyond scheduled group sessions, creating multiple communication channels to ensure participants felt supported. Many coaches shared their personal contact information and explicitly invited participants to reach out with urgent questions or concerns as “*some of [the] mentees, they sent emails 3 times, sometimes 4 times a wee.*” (NRMN_14). Several coaches emphasized the importance of communicating with participants outside of scheduled meetings. This ongoing support, provided through various channels such as email, Slack, or phone calls, was crucial for addressing urgent questions, offering continuous guidance, and ensuring participants remained on track with their grant writing and career development. This type of support also helped to reinforce the formal coaching sessions and allowed coaches to be responsive to the participants’ evolving needs.

Coaches tailored their availability based on participants’ needs as they recognized that quick, on-demand support was essential to maintaining momentum in grant writing, where even minor setbacks or uncertainties could stall progress. Some coaches described the importance of offering flexibility with meeting times, allowing one-on-one sessions when participants required additional guidance. By making themselves accessible in these ways, coaches fostered an environment where participants felt their questions were valued, helping to build participants’ confidence in the grant-writing process. This accessibility allowed participants to seek guidance when they faced immediate challenges, reducing delays in their progress and creating a sense of continuous support. Another coach noted how they adapted their support to the participants’ schedules and needs: “*I stayed in touch with participants who needed additional help*” (NRMN_05), indicating a flexible approach to coaching that extended beyond the confines of regular meetings.

#### Consider support beyond the coaching program

Several coaches continued to support participants even after the formal coaching sessions had ended. This ongoing relationship helped participants navigate challenges that arose after the program, providing them with a safety net as they advanced in their careers. Coaches described their efforts to stay in touch with participants beyond the structured program for continued coaching and support, particularly as participants worked on subsequent grant submissions or other career milestones. For example, one coach (NRMN_14) was asked for a letter of support for a separate proposal. Ongoing support beyond formal meetings played a vital role in the coaching process. Coaches who made themselves available outside of scheduled sessions provided participants with reliable guidance and reassurance, helping them overcome obstacles and achieve their goals. This continuous support was not only about being accessible but also about actively engaging with participants to ensure their success in grant writing and career development.

## Discussion

This qualitative study employed a semi-structured interview design to explore the experiences of established NIH-funded investigators serving as grant-writing coaches within the National Research Mentoring Network (NRMN) Strategic Empowerment Tailored for Health Equity Investigators (NRMN-SETH). Coaching is an emerging professional development strategy employed across the U.S., and this paper contributed to the conceptual definition of this activity and provided in-depth insights into the roles and processes of group-based, peer-supported grant-writing coaching. Coaches shared their reflections through guided interviews that elicited descriptions of their strategies, the challenges they encountered, and the techniques they used to foster participants’ growth. The study’s results revealed that grant-writing coaching in NRMN-SETH involved multiple facets, including personalized feedback, building confidence, managing group dynamics, and encouraging a supportive peer environment. Coaches emphasized the importance of individualized guidance, providing iterative feedback on grant sections such as specific aims and methodology, and adapting communication to fit participants’ needs. Another key coaching role was the creation of a safe and collaborative atmosphere that normalized participants’ challenges and allowed them to build both technical and social confidence. Additionally, structured approaches to group engagement and deliverable timelines fostered accountability and momentum. Notably, coaches reported that managing varying levels of experience among participants was sometimes challenging but was addressed through tailored support and additional coaching efforts. These findings provide a foundation for discussing the critical role that coaching plays in fostering the development of competitive grant proposals and, ultimately, in advancing the careers of early-stage investigators within the biomedical sciences.

Before discussing this study’s insights into the mechanisms and impact of grant-writing coaching, it is important to address its limitations. First, as a qualitative inquiry reliant on self-reported data from coaches, the study may reflect inherent biases related to participants’ retrospective perceptions and experiences. This approach, while beneficial for exploring in-depth personal accounts, limits the ability to assess outcomes through objective measures or standardized assessments of coaching efficacy. Another limitation is the absence of direct feedback from the program participants (i.e., early-stage investigators), whose perspectives could provide a fuller understanding of the program’s impact on grant-writing skills and career progression. Furthermore, the study’s findings may be influenced by contextual factors unique to NRMN-SETH, such as specific institutional and resource dynamics, potentially limiting the generalizability of the results to other institutions or populations. Future studies might address these limitations by incorporating mixed methods to triangulate the qualitative findings of this study with quantitative measures of participant engagement in mock sections and rates of proposal submission and award and include participant perspectives, to provide a more comprehensive evaluation of grant-writing coaching programs.

This study contributes to the growing conceptual discourse on structured, skill-specific coaching within career development, particularly in enhancing grant-writing efficacy among underrepresented early-career investigators. By delineating specific coaching mechanisms—such as iterative feedback, confidence-building, and managing group dynamics—the study provides evidence for the effectiveness of group-based, peer-supported professional development strategy, where coaching offers a task-focused, iterative learning approach that complements traditional mentorship [4, 14]. The experiences of the NRMN-SET coaches align with evidence suggesting that structured coaching targets to improve professional skill development, fosters self-efficacy [22], and complements institutional capacity to support early-stage investigators [32]. The evidence presented in this paper also supports the argument that group-based learning environments in coaching can catalyze the social support necessary for overcoming systemic barriers faced by underrepresented groups, thus addressing disparities in grant success and research productivity [17, 23].

The implications extend to institutional policy and faculty development programing, suggesting that embedding coaching programs within academic structures may increase grant-writing success rates for diverse populations, ultimately promoting inclusivity in biomedical research. Future studies could empirically test hypotheses that structured, iterative coaching leads to higher grant submission and success rates compared to traditional mentorship alone. A longitudinal, mixed-methods design could explore whether coaching impacts long-term career progression metrics such as independent funding attainment and publication rates. Additionally, further research could investigate the effectiveness of integrating peer-led components and mock review sections, building on findings that peer-to-peer feedback enhances learning [12, 31]. Testing these hypotheses could substantiate coaching as a pivotal intervention for developing research capacity among underrepresented scholars, informing broader workforce development strategies in academia.

The results of this study suggest several practical applications for structuring future grant-writing coaching programs within biomedical workforce development initiatives at minority-serving and low-resourced institutions. Implementing a structured, feedback-driven coaching approach could significantly enhance the grant-writing skills of early-career investigators, thereby improving their competitiveness in securing funding. Programs should consider adopting a multi-tiered coaching framework that includes one-on-one support, group-based sessions, and iterative feedback on specific grant sections, aligning with practices that help participants refine complex skill sets incrementally [4, 14, 34]. To maximize impact, institutions could also facilitate peer-support networks and mock study sections, which foster collaboration and normalize common challenges in grant development, enhancing participants’ confidence and resilience [12]. For sustainability, securing institutional buy-in to provide protected time and resources for participants is essential, allowing them to prioritize these coaching activities amid other academic responsibilities. These strategies, grounded in the findings of this study, offer actionable frameworks for creating supportive environments that can empower underrepresented scholars to navigate the competitive landscape of biomedical research funding.

In conclusion, this study highlights the multifaceted role of grant-writing coaches in supporting early-career investigators from underrepresented groups within the NRMN-SETH program. The findings demonstrate how targeted, individualized feedback, confidence-building strategies, and a collaborative group environment foster essential skills for successful grant writing. By integrating these insights into structured programs at minority-serving and low-resourced institutions, coaching can serve as a powerful tool for advancing research equity in the biomedical workforce.

## Supporting information

10.1017/cts.2025.10080.sm001 Levites Strekalova et al. supplementary material Levites Strekalova et al. supplementary material

## Supplementary material

The supplementary material for this article can be found at https://doi.org/10.1017/cts.2025.10080
