# Supplementary material for: Exploring grant writing coaching and its role in professional development of health equity investigators: A qualitative study
Source: J Clin Transl Sci. 2025 Jul 1;9(1):e178. doi: 10.1017/cts.2025.10080 (PMC12444689; doi:10.1017/cts.2025.10080)
Supplement: Levites Strekalova et al. supplementary material [file S2059866125100800sup001.docx]

**Appendix A. Interview Guide**

[Intro]

Please describe your overall role and experience with the NRMN program.

[Needs]

In your opinion, what are the key gaps in grant writing support that the NRMN program aimed to fulfill?

Were these needs met?

What are the needs and gaps that remained unfulfilled?

What was your experience interacting with the NRMN program coordinators and staff?

What worked well?

What would you change?

[Processes]

How did you structure your interactions with the program participants?

What worked well, and what would you change?

What processes of this group-based program were most effective?

What processes did not work well?

To what extent did participants in your group engage with the coaching process?

To what extent did participants in your group engage with the peer group?

What was your experience with mock study sections?

What do you think worked and didn't work in terms of scheduling, the timeline, the reviewers, etc.?

Did you attend the Mock Study Section with one of your mentees or get their feedback about the process?

To what extent would you consider the mandatory Mock Study Section to be helpful?

[Outcomes]

How would you assess the outcomes of this program?

What did the program was able to accomplish?

Where did the program fall short?

[Structures]

What, if anything, stands out about the NRMN approach to grant writing mentoring and coaching?

If someone were to replicate the NRMN experience at another institution, what infrastructure would they need?

From your perspective, what infrastructure (people, technology, etc.) is key to this grant-writing coaching program and its capacity to support early-stage investigators?
